# ‘It’s Not Like in the Films’: Bereaved People’s Experiences of the Deathbed Vigil

**DOI:** 10.1177/00302228221133413

**Published:** 2022-10-14

**Authors:** Glenys Caswell, Eleanor Wilson, Nicola Turner, Kristian Pollock

**Affiliations:** 1 Independent Social researcher, 6123 University of Nottingham, Nottingham, UK; 2 NCARE, School of Health Sciences, 6123 University of Nottingham, Nottingham, UK; 3 Department of Oncology and Metabolism, Medical School, 7315 University of Sheffield, Sheffield, UK

**Keywords:** deathbed, dying, family, moment of death, vigil

## Abstract

This paper explores how people enact and experience the deathbed vigil when someone close to them is dying. It draws on qualitative interviews with 34 bereaved people carried out as part of a wider study exploring public perceptions of death and dying. Participants were aware of the expectation that they would attend the deathbed and did their best to do so. Findings are reported using four themes: gathering, enacting the deathbed vigil, experiencing the deathbed vigil and moment of death. Participants’ experiences varied. Some families kept vigil as a group, while others established a shift system or waited alone. Activities at the bedside included reading to the dying person, talking amongst themselves, sharing memories, saying goodbye. The covid-19 pandemic highlighted families’ wish to accompany their dying relatives.

## Introduction

Dying is a relational experience affecting not only the person who dies, but also those around them, including family and friends (Borgstrom et al., 2019). Professional healthcare leads the management of dying in Britain, yet those who are approaching the end of life spend most of their time at home, supported by their family (Smith, 2019). End-of-life care policy in Britain prioritises the wishes of dying individuals, but most people make decisions as members of a familial group, however that group may be constituted (Borgstrom et al., 2019; Department of Health, 2008). The term family will be used here to refer to those closest and most important to the person who is dying, whether that be blood relatives, wider family or friends.

Dying is a key life transition for humans; it impacts everyone, both as a mourner and, ultimately, as someone who must face their own mortality. All societies have processes to manage dying and death, but the form of these processes is context dependent (Davies, 2002). The deathbed vigil, which takes place when it has been acknowledged that a person is dying, follows a familiar cultural script in Britain. This script, which clearly but succinctly articulates cultural norms, carries with it the expectation that no one should die alone and is dominant in shaping family behaviour towards the end of life (Caswell, 2020; Richards & Krawczyk, 2019). Typically, family members and friends will accompany the dying individual during the last days or hours of their life. The presence of people who are emotionally close to the dying individual may be supplemented by professionals who bring expertise to the bedside, and endeavour to ease any suffering experienced (Kellehear, 2013). This paper draws on interviews with bereaved people to explore how they enacted and experienced the deathbed vigil of their family member.

The paper begins by clarifying how the term vigil is used, before moving on to consider the British deathbed vigil. This will be followed by a description of the research study and then the findings, which are presented using four themes: gathering, enacting the deathbed vigil, experiencing the deathbed vigil and moment of death. A discussion will draw together elements of the findings to consider how the deathbed vigil is enacted and how families use that time to mark the significant occasion they are experiencing.

## The Deathbed Vigil

The noun vigil has several meanings, including ‘a devotional watching’, and ‘an occasion or period of keeping awake for some special reason or purpose’ (Friedrichsen, 1973). Use of the term vigil suggests that the deathbed is perceived as important, with special, possibly sacred, purposes involved in its enactment. Typically, the deathbed represents a time when family members accompany their dying relative, while watching and waiting (Kellehear, 2014). The term vigil is used in this paper in relation solely to the deathbed, rather than other kinds of vigil which might be maintained, such as watching over a dead body. It is used here as a specialist term and is not one which families would necessarily use themselves, despite their commitment to accompanying their dying relatives.

The deathbed vigil in Britain is embedded within cultural consciousness, so that there is a cultural script which serves as a normative guide informing people that they should accompany those who are dying and about whom they care (Kastenbaum, 2000). The image of the deathbed is a familiar one from different stages of history, from mediaeval paintings to Victorian fiction and 21^st^ century soap operas (Aries, 1985; Caswell, 2020; Mullan, 2014). Illustrations of the deathbed vigil have sometimes romanticised it, presenting what may be a turbulent death as tranquil (Jalland, 1999: 234).

Once it has been acknowledged that the person is close to the end of their life the vigil may begin. During the mediaeval period in Britain it was expected that the dying person would recognise their approaching death and make their own preparations (Aries, 1974). In the 21^st^ century, however, it is more likely that a professional will make the judgement that someone is dying, and inform their family (Caswell et al., 2015). This is not always an effective way of enabling relatives to reach the bedside before the person has died. One study found that approximately half of bereaved people were not called in time to be with their person before they died (O’Sullivan et al., 2021). The composition and purpose of the deathbed vigil has changed over time. A priest was once necessary to ensure that the dying person had been absolved of their sins, and the family and friends also had a role to play in praying for the soul of the dying person (Pasulka, 2018). In the 16^th^ and 17^th^ centuries there may have been a lawyer present to ensure that a will was drawn up and dealt with (Hallam, 1996; Hallam et al., 1999). At all times, a female nurse may have been present to care for the dying person’s body. In the 20^th^ and 21^st^ centuries there may be health or social care professionals on hand, whose role is to diagnose imminent death and ensure that the dying person is as comfortable as possible.

Family and friends present may play a supporting role in the care of the dying person’s soul or body. However, the primary reason for family to be at the deathbed is to enact their relationship with the dying person and express the closeness of their bonds. Accompaniment, sometimes expressed as companioning, is about having a physical presence with the person who is dying (Bowman, 2011). Approximately 10% of people who die do not have family or friends to be with them (Kristensen et al., 2021). A range of organisation, projects and roles have been established which try to fill the gap, such as end of life doulas, soul midwives, hospice and hospital volunteers (Bloomer & Walshe, 2020; Burbeck et al., 2014; Krawczyk & Rush, 2020).

The vigil has been considered an opportunity for the dying person to say their farewells (Kellehear & Lewin, 1989), and may also give families the chance to ‘…protect and advocate for the dying person…’ (Kellehear, 2013: 109). Bereaved study participants in Ireland spoke of the vigil as a significant time of farewell, through ‘…its reciprocity or mutuality’, suggesting its importance to all parties (Donnelly & Battley, 2010: 98). Dying is often described as medicalised, with professional institutions and practitioners having an over-influential role in decision making and orchestrating the end of life (Koksvik et al., 2020). The vigil is one possible way of attempting to reclaim dying on behalf of both the family and the dying person, and as a way of affirming bonds in readiness for the post-death period (Valentine, 2007).

The moment of death, with family members accompanying the dying person, has always been considered significant (Donnelly & Battley, 2010; Donnelly & Donnelly, 2006; Valentine, 2007). Some of Valentine’s (2007) bereaved research participants missed this moment, either because they had left the bedside, or because they did not realise that the person had died. Missing the moment caused some participants to express feelings of ‘…disappointment and guilt…’ (Valentine, 2007: 226).

The importance of the deathbed vigil is highlighted through attention drawn to its absence during the covid-19 pandemic. Contact with people who were dying at home was reduced for both professionals and family members, which may have been both distressing and solitary for some people, whilst simultaneously the number of home deaths increased (O’Donnell et al., 2021). Hospitals and care homes were legally required to stop families visiting their relatives, even when they were dying. Some institutions developed innovative ways of trying to obviate the negative effects of such absences by using digital technologies and some endeavoured to ensure that a member of staff prioritised spending time with dying patients (Hanna et al., 2021). Vigils with people who were dying at home and who lived alone were also curtailed during the period of pandemic restrictions (Schloesser et al., 2021). There is evidence to suggest that separation from family can increase the likelihood of a patient enduring delirium or anxiety, and it is suspected that the family may experience a poorer than otherwise bereavement outcome (Downar & Kekewich, 2021).

The deathbed vigil thus has a long history in Britain, and it carries the expectation that family members and friends will accompany the dying person during their final days and hours. Despite the commitment to maintain a vigil for dying relatives, very little is known about what happens during these occasions, or how they are anticipated and experienced by relatives; this paper thus makes a contribution to our knowledge about an important aspect of the social management of death and dying.

The literature offers a range of suggestions as to what the vigil can accomplish, and the aim of this paper is to explore how bereaved people enacted and experienced the deathbed vigil. The next section describes the methods used in the research.

## Methods

This paper draws on interviews with bereaved family caregivers, and is one aspect of a mixed methods, qualitative study with three phases. The first phase comprised a series of deliberative discussion groups with members of the public to discuss different aspects of death and dying over a number of sessions. The second phase was made up of interviews with bereaved people and the third phase was composed of interviews with people who had been given a diagnosis of terminal illness. The study aim was to gain insight and understanding into the perspectives of members of the public about issues around death and dying. The study was funded by Marie Curie.

### Interviews With Bereaved People

As part of phase two, 34 bereaved individuals took part in a one-to-one semi-structured interview. Twenty-six were recruited through adverts in a range of places including twitter, Facebook and Call for Participants, as well as in community newsletters and magazines in the East Midlands, with a further eight bereaved individuals recruited through two independent hospices.

Interviews took place between October 2019 and February 2021. Prior to the March 2020 pandemic lockdown participants were offered the choice of being interviewed face-to-face or via video link or phone. 14 interviews were completed before lockdown began; three via video link, one by phone and the other 10 were face-to-face. From 23^rd^ March 2020 onwards potential participants were offered the option of either a telephone or online interview and eight bereaved people opted for video link, while 11 chose a telephone interview and one decided to take part through a series of email exchanges with a researcher.

Interviews were conducted by one of three researchers, all of whom are social scientists, using a topic guide. The interviews began by asking about the experience of a specific bereavement, using this as a starting point to explore other experiences of dying and to engage with participants’ views about death as a topic. Most participants spoke about one bereavement, but eight described more than one. Table 1 gives basic information about participants, with pseudonyms used in place of real names. All interviews, with participant consent, were audio-recorded. Recordings were transcribed by professional transcriptionists and data were analysed using NVivo12© as a data management tool. Analysis was an iterative process based on the constant comparative method, from the desire to ground the analysis in the data (Charmaz, 2006). Each transcript was coded by at least two members of the research team and regular discussions were held about the coding scheme as it developed.

**Table 1.** Participant Characteristics.

Pseudonym	Age	Sex	Deceased relationship to participant	Deceased diagnosis	Place of death
Jan Ferguson	57	F	Father	Dementia	Care home
Heather Smith	48	F	Mother	Ovarian cancer	Hospice
Carol Milligan	50	F	Mother	Renal failure	Home
Susan Crawford	62	F	Mother	Pneumonia	Hospital
Doris Taylor	82	F	Husband	Multiple co-morbidities	Home
Annie Draper	64	F	Mother	Liver cancer	Home
John Clark	65	M	Father-in-law	Cardiac arrest	Home
			Stranger – mountain rescue	Accident	Outdoors
Naomi Jarvis	40/50s	F	Mother	Stroke	Hospital
Mike Burt	56	M	Wife	Cervical cancer	Hospice
			Mother	Unknown	Hospital
Felicity Burgess	65	F	Mother	Heart failure	Care home
			Husband	Multiple sclerosis	Hospital
Paula Stokes	63	F	Father	Colorectal cancer	Home
			Mother	Anorexia	Care home
			Friend	Pancreatic cancer	Home
Carrie Nolan	26	F	Grandmother	Kidney failure	Home
Lynn Fraser	19	F	Grandmother	Stroke	Hospital
Timothy Brentwood	46	M	Father	Metastatic mouth cancer	Home
Rob Haywood	79	M	Wife	Cancer	Hospice
Mark Hill	73	M	Friend	Mesothelioma	Dignitas
Jess Lewis	66	F	Friend 1	Bone cancer	Unknown
			Friend 2	Mesothelioma	Dignitas
			Friend 3	Progressive supranuclear palsy	Dignitas
			Grandmother	Pneumonia	Hospital
Annabel Grant	50	F	Father	Cancer	Hospice
Sheila Moore	81	F	Husband	Heart disease	Hospital
			Friend’s child	Pneumonia	Unknown
			Friend	Multiple sclerosis	Hospital
Sarah Swain	68	F	Friend	Brain cancer	Nursing home
			Uncle	Frailty	Nursing/care home
Helen Bryant	56	F	Mother	Not stated	Home
Maureen Cooper	68	F	Mother	Dementia	Nursing home
Fiona Ross	59	F	Friend	Cancer	Hospice
Amy Watkins	67	F	Sister	Gynaecological cancer	Home
Maria O’Sullivan	69	F	Mother	Pneumonia	Care home
			Father	Sepsis	Care home
Bruce Irving	72	M	Wife	Bowel cancer	Hospital
Trevor Murray	79	M	Wife	Lung cancer	Home
Imelda Kelly	62	F	Husband	Prostate cancer	Hospital
Rachel Norris	63	F	Daughter-in-law	Bowel cancer	Hospice
Peter Quinell	75	M	Wife	Pancreatic cancer	Hospice
Hannah Porter	73	F	Husband	Head and neck cancer	Home
Les Vaughan		M	Wife	Cancer	Hospice
Diane Hamilton	67	F	Husband	Metastatic bowel cancer	Hospital
			Mother	Stroke	Home
Oscar Reed	66	M	Wife	Cancer	Home

### Consent

All participants were given an information sheet about the study to enable them to come to an informed choice about whether to take part, and all gave consent to participate. For face-to-face interviews this involved the signing of a consent form. For virtual and phone interviews consent was taken through the completion of consent forms by email, through the post or through verbal consent being audio-recorded. In addition to consent forms, all participants were asked to complete a form giving basic demographic information about themselves.

### Ethics

Approval to recruit participants through social media and community groups was obtained through the University of Nottingham, Faculty of Medicine and Health Sciences research ethics committee, with approval given on 8th October 2019, REC reference 385–1909. Approval for recruiting through the NHS and independent hospices, was sought through the Health Research Authority, which was given in January 2020, following review by a research ethics committee; REC reference 19/EM/0327.

The main issue of ethical concern when interviewing bereaved people is that discussion of such a sensitive topic as the death of someone about whom they cared could be distressing. The information given to potential participants about the study asked them to consider this carefully before deciding to take part. All members of the research team are experienced in conducting end of life research and were alert to signs of distress. Participants were assured that they could pause or end the interview at any time; while there were pauses, none of the interviews were ended.

## Results

Findings presented here draw on data from interviews with 34 bereaved people. In total participants gave detailed accounts of 47 deaths, of which two were sudden and unexpected, but the remainder were after a period of illness. Of the 47 deaths discussed, 14 took place at home, 11 in hospital, eight in a hospice and eight in a care home. One sudden death occurred outdoors, and three deaths discussed took place at Dignitas in Switzerland. In two instances information about place of death was not discussed.

Although some of the interviews were conducted during the period of the covid-19 pandemic, none of the participants spoke of deaths which occurred under pandemic restrictions. Out of the 47 accounts, 27 deaths were attended by the participant, 18 were not present and in two cases attendance was not reported. In most cases participants had wished to be at the deathbed but were sometimes prevented because they did not realise how close to death their person was and no one told them, because there was insufficient time to enable them to travel, or because they had caring responsibilities which prevented them being present. Some participants were distressed by missing the death of their person, although in one case the participant was pleased to have missed her husband’s dying.

A main concern for all participants was that their relative should be well cared for at the end of life, so that they were comfortable and pain free. In most cases they felt a strong desire to be with them throughout their dying, without necessarily being able to articulate why this was so important. Felicity, for example, when asked what made it so important that she should be with her dying husband, was unable to pinpoint reasons saying, ‘Oh golly, oh had to be there.’

None of the participants, however, referred to this process of accompaniment as keeping vigil; rather they spoke of gathering, coming together or sitting with the dying person. Despite their knowledge that they were expected to be present, and the commitment they felt to be there when their family member was dying, not all participants knew what they were supposed to do when they reached the bedside of their dying relative or friend, so that they found themselves making it up as they went along. For some participants spending time with the dying person was a positive experience which they valued at the time and in retrospect. However, this was not always the case and some participants described either a mixed experience or one which left them with difficult memories to deal with.

We have identified four themes to explore participants’ experiences. These themes are: gathering, enacting the deathbed vigil, experiencing the deathbed vigil and moment of death.

## Themes

### 1) Gathering

Once they became aware that the person’s death was imminent, families gathered and waited, sometimes for hours, sometimes for weeks. In some cases, they were advised by a healthcare professional, as was Maureen’s experience: ‘the nursing home rang me up early in the morning and said we think that she’s dying…So I managed to get there’. However, staff might not contact family members early enough. Maria, for example, said ‘So I wasn’t contacted until 8 o’clock the next morning which is just about when my mother died and I… got there too late.’ A new senior care worker had not realised that she should check the communication book to see that Maria wished to be informed when her mother was dying.

Sometimes a member of the family would recognise or suspect that their relative was approaching the end of life and take responsibility for calling their family together. Jan, for example, said ‘So it was me that gathered all the family… to start saying goodbye to (their) granddad’. Paula was not present when her father died although, as she said, ‘I really wanted to be there when he died.’ However, this was not possible because family dynamics meant, Paula said, that ‘I wanted to be but was not welcome at that point.’

The gatherings described by participants varied. In some instances, families set up shift systems, so that there would always be someone with their dying relative. Carrie, for example, when her grandmother was dying at home, said ‘we were shifting it so that one person would leave at a time to go home, have a shower, get some more clothes, whatever.’ Only Felicity and her brother were present to sit with their mother as she was dying, and in some cases there was only one person keeping vigil, as Susan said of her mother dying in hospital, ‘I was there for most of the time, because there’s only me really’.

Relatives were not always informed in good time by health professionals about the imminence of death, making it difficult for them to step away from their daily responsibilities to establish a vigil. Sometimes this might be because it can be difficult for professionals to predict that death is imminent, but also sometimes there was a lack of communication with, or awareness of, family preferences. When families were able to gather to sit with their dying person the length of time for which this was required varied. Susan’s mother, for example, was recognised to be dying for 3 weeks, but Maureen had only a few hours to be with her dying mother. When a vigil extended too long it could become beyond the resources of the individual or family to manage, given the other responsibilities in their daily lives.

### 2) Enacting The Deathbed Vigil

Typically, wherever the place of death, participants expressed a wish to say goodbye. Despite the existence of a cultural norm to keep a vigil, this is not necessarily accompanied by the knowledge of what to do, and guidance towards conventional practice is lacking. Sometimes individuals or families would sit quietly beside their relative, unsure what else they could do. Other families engaged in activities at the bedside such as talking amongst themselves and to the dying person even if they did not appear able to hear, sharing memories, telling stories and reading to the dying person.

#### Home Death

14 of the deaths discussed took place in the dying person’s home, although research participants did not necessarily live with them. This meant that family members were taking responsibility for the person’s physical wellbeing, as well as having a perceived duty to maintain a vigil. Most participants in such cases talked about trying to ensure that the dying person was not left alone. Timothy, for example, stayed at his parents’ home when his father was dying. His mother was the primary carer, and he felt that ‘all I could do is sit and watch’, although he was pleased because ‘I managed to say a few things to my dad that I needed to say and we both understood each other’. Timothy had experienced other family deaths and had been left feeling that there were things he should have said, but this was not the case with his father’s death.

Carrie came from a large family, who gathered at her grandmother’s home when they realised that she was dying. Carrie said:

*We were all going in and taking turns just to sit with her, hold her hand, talk to her. She wasn’t very responsive at this point… but you take comfort in sitting and talking to her and whatever.*

Sitting with, talking to and watching over the dying person were key activities for those who maintained a vigil at home.

#### Hospital Death

The way in which a vigil could be kept was constrained by the environment for families whose relative was dying in hospital, with differences in setting of ward or side room. Their relative was likely to be in a ward amongst other patients with only a thin curtain to offer an illusion of privacy. Families could thus feel the need to be quiet, and not to take up too much space.

Bruce felt fortunate that his dying wife was in a side room, which allowed the family privacy to sing hymns:

And we just sat round the bedside and sang hymns… Singing hymns that had depth of meaning was important. And we all sang together.

Imelda’s husband was not so lucky, as he was dying in a shared space. Imelda was, however, able to arrange for her husband’s pet dog to visit him in the hospital one last time:

So we managed to sort it out with the sister on the ward for the dog to come up and so the dog came up on the Friday…He touched her. We put his hand, I think he knew she was there.

#### Care Home Death

There is an expectation that people who live in a care home will have a room to themselves (Department of Health, 2003), suggesting greater privacy than may be experienced in the hospital setting. When family members were able to be present they sat, and talked with, their dying relative.

When Maria’s mother died there was no gathering of the family, because they did not know when it was time to gather. This influenced the family’s behaviour later, when their father was dying, and they took care to ensure that some of the family were present. Maria said:

And my brothers visited, so I had somebody there for each of those days and we sat at his bedside and chatted and we drew him in to the conversation as much as we could, but he was sleeping a lot.

Jan, whose father also died in a care home, said, ‘We were like talking and laughing and joking but being mindful of my dad who had no response, there was no response all the time that we were there.’

#### Hospice Death

From participant accounts, hospices were better placed to support the families of dying patients. It was not unusual for the patient to be in a private room, nor for a bed to be brought in for a family member to sleep beside the dying person. For example, Mike’s wife died in a hospice, which provided a room large enough to hold a second bed for him to sleep in, as well as other people to visit and ‘on the actual night she passed, I think there was about six or seven of us in the room.’

Annabel’s father also died in a hospice and she and her family sat with him and said their goodbyes. She said, ‘I told him everything I needed to tell him, and I told him not to be scared, and I was just holding his hand.’

Participants whose relative died in a hospice described processes of spending time, talking with and waiting beside their dying person.

### 3) Experiencing The Deathbed Vigil

For some participants spending time with their dying relative was a positive experience, but for others the experience was less clear cut, with some aspects perhaps good but others less so. It was also the case that for some participants the experience of their relative’s dying was difficult, particularly in relation to physical symptoms exhibited by the dying person.

#### A Positive Experience

Felicity found it comforting when she looked back to think that she had been with her mother when she was dying in a care home:

*So me and my brother just sat with her, and she was unconscious, but we were just talking to her all day until she died, yeah so that was nice to be with her and very comforting.*

Jan took charge when she believed that her father, who lived in a care home, was approaching the end of his life, and had summoned her family to sit with him. She felt that this was a significant experience:

*My brother came just, me and my mum were there and then my brother turned up and I knew death was imminent. I just knew, we were like, any second, any time, any minute, and my mum just started to chat about absolutely nothing. And I knew that was just avoiding the subject…Because for me it was like a really sacred experience that was going to happen and she just wanted to chat about nothing at all. And my brother, bless him, he did respond and said shall we just be quiet mum. And she went oh OK.*

When a death was perceived to be peaceful this could contribute to the participant having had a positive experience, as Annabel said of her father’s death in a hospice:

*But I honestly do feel he was very peaceful, he was surrounded by all of us…my older brother wasn’t there but he came just after he died. So we were all there…We’d all said our goodbyes.*

#### A Mixed Experience

Annie cared for her mother when she was dying at home and had night sitters to support her. She said:

*I was in bed and they knocked on my bedroom door…they said I think your mum’s going to die soon… So that was that…. It’s not like in the films, where it’s all nice and serene and, or in the old pictures where everybody was around the bed. It’s not like that.*

#### A Difficult Experience

Imelda’s husband died in hospital, and she was left with difficult memories, saying, ‘It’s just all these pictures of him in my head because I remember everything, it’s just horrible.’

Susan found the experience of her mother’s hospital death difficult to handle, saying:

*She wasn’t moaning or anything like that. But the breathing for 3 weeks… they said that she’s not in distress; it’s just a natural bodily reaction. But processing that, you know, logically you can understand it, but when you’re hearing it and you’re hearing it for 3 weeks, sometimes it was just too much, which is why I wanted to escape sometimes.*

For Heather the experience of her mother’s death in a hospice was traumatic, with sounds and smells which stayed with her over time:

So I went behind the curtain and just, she was lying with her mouth open. I thought I was going to remember that noise for the rest of my life, and I can’t remember it anymore, which is really good.

The location of the death did not appear to be linked with the nature of the experience in a significant way: good and bad experiences were reported in all settings.

### 4) Moment of Death

Being present at the moment of death was thought by most participants to be important. Jan called being with her father when he died in a care home ‘a sacred experience’, although for some it was more of an anti-climax, which might even pass unnoticed. Several participants described how they experienced being present for the moment of their relative’s death.

Heather talked about how she and her siblings sat with their mother ‘… giving her permission to pass away’, saying that she felt ‘privileged to have experienced her last breath’.

Helen’s mother died at home, having expressed a wish to die with her husband holding her hand. Helen was keen that her mother should not be left alone:

*I sat with her and…I kept thinking oh she’s going to go in a minute, but she didn’t. And then dad said oh I’m going to get up at about eight o’clock, and I said oh no don’t get up yet, just stay with mum for 10 minutes, I’ll go and have a quick shower, and then I can sit with her…And within 2 minutes of me going out the room she died.*

How many people were present depended, among other things, upon the configuration of the family and the way in which the family’s efforts at organisation evolved. Maria was alone with her father when he died, although her brothers had spent time with him on previous days. When Carrie’s grandmother died there were several people present. She said:

*My mum, my auntie, my uncle, my granddad, me and the carer and one of the current carers were in the room, and she just stopped breathing really calmly, really peacefully.*

There had been many visitors during the day, but at the moment of her daughter-in-law’s death in a hospice only Rachel and her son were present. She said, ‘And I was watching her. And in that split second she stopped breathing.’

Not all participants were present when their relative died and there might be, as Naomi said, ‘… millions of reasons why somebody’s on their own (when dying)’. Rob’s son missed the moment of his mother’s death:

*My son were sitting with her and (my son) had been up all night the previous night with her and I said to him, lad you’re dead on your feet, you really need to go home and get some rest...And I sat there…eventually her breathing was getting different and it just stopped …the sad thing was then I’d sent my son home and he was not there at the actual time that it happened. So that was a bit upsetting for him.*

Diane missed being with her husband when he died because she had to leave the hospital to look after her mother. She said:

*I had to leave my husband, who was at least safe in hospital, to come home and relieve my brother and look after my mum. And I’d only been home about an hour and I got a phone call from the hospital to say that he’d suddenly deteriorated and could I come. So I had to phone my brother again…but I got there 10 minutes after he died.*

For a minority of participants, the notion of being present when their relative died was undesirable. Timothy’s sister arrived at the family home after their father had died and Timothy was concerned that she would be upset about this. However, he learned later that she had not wanted to witness their father’s death. Sheila spoke about the death of her husband:

*But anyway they (the nurses) had sorted him all out and freshened him up and then left him. And when 5 minutes later they came back he had died. So I think he had just slipped away*.

Sheila went on to say that, for herself:

I don’t know, but in a funny way I’m not sure that I do want a cluster of people around my bedside; I’d rather just slip away by myself.

Participants’ experiences of the deathbed vigil were diverse; most lacked personal experience of keeping vigil and did not know what to expect at the moment of death. They were, to an extent, making it up as they went along.

## Discussion

Participants expressed a need to spend time with their dying relative and to be present when they died. This was not always possible, for participants relied on health or social care professionals to advise them that their relative was close to death. This had mixed results, so that sometimes participants were contacted too late and did not get to spend time with their person. Some participants were unable to take time out from other activities in their day to day lives, making it impossible for them to gather and keep vigil with their dying relative.

Once family members had gathered at the bedside, whether as a large or small group, they did not know what they were expected to do, and the location of the dying seemed not to make a difference. There were constraints when the person was dying in an open hospital ward, such that participants were aware of the lack of visual and auditory privacy which was possible in other settings. The interactions which took place seemed mundane, yet were significant as people could meet up and spend time together. This gave them the opportunity to consolidate and renew existing bonds, thus demonstrating the nature and extent of enduring ties following the person’s death. In some cases participants told the dying person things which they felt they needed to, thus completing unfinished business with their relative. Participants spoke to the dying person, or read to them, even though he or she seemed unaware and was either sleeping or unconscious. The dying person was thus generally perceived as a passive actor in the performance of family relations, and those relations did not necessarily change their nature just because a family member was dying.

Participants described varied experiences at the deathbed vigil. For some it was positive and they valued the opportunity to spend time with their dying relative. For others, however, it was a traumatic experience and they were left with long-term memories of the visceral nature of the dying process. This highlights the tension that exists between the idealised tableau of the good death, with family members keeping vigil at a quiet and tranquil deathbed, and the reality of dying which may be very different, involving noise, unpleasant sights or smells and a dying person experiencing negative symptoms such as pain or anxiety. Familiarity with the cultural script leads people to have high expectations of the vigil, but feeling the obligation to be present did not stop the vigil from being uncharted territory for participants who found the reality both shocking and hard to deal with. This disjuncture between expectation and experience also points to a difficult contrast between the sacred and the profane aspects of the vigil. Jan was unusual in describing her experience when her father died as sacred, yet she was able to orchestrate the vigil to meet her needs and expectations, having decided that the time was right to summon her family to sit vigil. Most participants, however, lacked the experience, knowledge and opportunity to take control of the vigil.

Consistent with previous research, participants believed that the moment of death was of particular importance (Donnelly & Battley, 2010; Donnelly & Donnelly, 2006; Valentine, 2007), although not all participants were present at the time of death. Some were content not to have been, although most participants either were, or wished they had been, present. One consequence of the norms around keeping a vigil is that this could make it hard for people to reject a wish to be present or to express their misgivings, and this may be linked to the role of the vigil in regulating the social management of dying. In a small number of cases, however, participants did not realise that the death had occurred, despite being present at the time.

Participants did not talk about keeping vigil, but of gathering and waiting, typically to say goodbye (Donnelly & Battley, 2010; Kellehear & Lewin, 1989). The number of people at the bedside varied, but the vigil was maintained, whether one person or a group were present (Kastenbaum, 2000). Different activities were undertaken including talking, laughing, eating, reading, and the singing of hymns. This was consistent with Kellehear’s (2013) conception of the vigil as comprising activities similar to those of the wake, and to the notion of saying farewell to the dying person (Donnelly & Battley, 2010; Kellehear & Lewin, 1989). However, there were also instances when the family who were present sat quietly engaged, perhaps, in watchful waiting and being present for the dying person.

The uncertainty which participants expressed about what they should do during the deathbed vigil illustrates that they recognised this to be a special occasion and one where they should show an awareness of that.

## Conclusion

The cultural script which promotes accompaniment of those who are dying continues to be strong. The deathbed vigil, as enacted and experienced by our participants is a social event, reflecting a continuity with vigils of the past, with the addition of contemporary mores and family practices. Participants did not use the term vigil to describe their accompaniment of the dying person which suggests, perhaps, unawareness that they were continuing a long-established practice.

Each participant described the ways in which they portrayed their version of family and how this translated in terms of activities. There appears to be a dissonance between the everyday nature of these activities and the reality of the death that was occurring while they were going on. In many ways the process at the end of a life seems mundane, yet the outcome is far from being routine or banal for those who are bereaved. As a social, rather than medical, event, the deathbed vigil is not just for the dying person, it is also for the living and can be enacted as such.
